# Dismantling the status quo: promoting policies for health, well-being and equity: an IUHPE2022 prelude

**DOI:** 10.1177/17579759211019214

**Published:** 2021-06-16

**Authors:** Brittany Wenniserí:iostha Jock, Carole Clavier, Evelyne de Leeuw, Katherine L. Frohlich

**Affiliations:** 1 Centre for Indigenous Peoples’ Nutrition & Environment (CINE), McGill University, Montreal, Canada; 2 Département de Science Politique, Université du Québec à Montreal, Montreal, Canada; 3 Centre for Health Equity Training, Research & Evaluation (CHETRE), UNSW Australia Research Centre for Primary Health Care & Equity A Unit of Population Health, member of the Ingham Institute, Sydney, Australia; 4 École de Santé Publique (ESPUM) et Centre de Recherche en Santé Publique (CReSP), Université de Montreal, Montreal, Canada

**Keywords:** advocacy (including media advocacy), collaboration/partnerships, determinants of health, empowerment/power, equity/social justice, global health/globalization, health promotion

## Abstract

The next international gathering of the global health promotion family will be in Montreal, in May 2022. The 24th IUHPE conference is themed ‘Promoting policies for health, well-being and equity’. Conference organizers have decided to transcend the ‘usual suspects’ rhetoric and frame a conference program that truly challenges these key notions for health promotion. In this contribution, members of the Canadian National and Global Scientific Committees reflect on the state of play and the opportunities ahead. We propose three themes as follows: (a) breaking news (the promise and opportunities for disruptions and tipping points, whether from pandemic health challenges, climate change, geopolitical shifts, social unrest or technological promise); (b) breaking free (from world-views that favor only market solutions, divisions between North and South, toward emancipatory decolonizing practices and knowledge systems); and (c) breaking through (disciplines, silos, boundaries and identities engrained in our practices and understandings for innovation.)

Health promotion as a field of practice and scholarship continues to evolve. As a global movement, the health promotion community embraces many voices. There is a challenge, however, in continuing to strike a balance between elegant and advanced techniques and approaches on the one hand, and hands-on and often acute health issues on the other. Global conferences of the International Union of Health Promotion and Education have – most of the time – successfully walked this fine line. The next international gathering of the global health promotion family will be in Montreal, in May 2022. The 24th IUHPE conference is themed ‘Promoting policies for health, well-being and equity’. Conference organizers have decided to transcend the ‘usual suspects’ rhetoric of striving for equity in health (through discussions, for example, of issues such as the Social Determinants of Health, and Health in All Policies), to instead look to the root causes of health inequities and their structural determinants, including those that are political, economic, environmental, cultural and social. The IUHPE2022 scientific committees are framing a conference program that truly challenges the foundations and directions for policy with regard to health, well-being and equity for health promotion. In this contribution, members of the Canadian National and Global Scientific Committees reflect on the state of play and the opportunities ahead.

Health promotion remains ‘... the process of enabling individuals, groups and communities to increase control over, and to improve, the determinants of their health. To reach a state of complete physical, mental and social well-being, an individual or group must be able to identify and to realize aspirations, to satisfy needs, and to change or cope with the environment’ (1). Our community positions itself as a positive social movement; one that engages with optimistic views of what determines human and ecological, indeed planetary, health and well-being. We also strive for social justice and the reduction of all inequities (social, ecological, cultural, or by any other parameter) that adversely influence health.

As we write this commentary, however, coronavirus disease 2019 (COVID-19) is wreaking havoc on populations and economies, revealing not just our fragility in the face of new infectious diseases, but also how much climate change, systemic social and racial inequities, and the failures of our political and economic systems have to bear on our collective well-being. This has not just been deemed an epidemic of global proportions (a ‘pandemic’) but indeed a ‘syndemic’ – a systemic coalescing of health and social events that exposes critical fault lines across the world (2). Horton, *The Lancet* editor, has justifiably stepped away from the more epidemiologically driven notion of ‘syndemic’ as originally proposed by Singer (3). The tragedy of the COVID-19 syndemic is not just the inequitable impact of a series of disastrous (clinical) co-morbidities, but also the effect of a world permitting for the deaths of hundreds of thousands in superficially wealthy and powerful nations. The syndemic is also the result of a perverse disregard for large swaths of disadvantaged populations precariously keeping neoliberal economies afloat. It highlights, for instance, that we undervalue and underpay millions of essential workers. We are witnessing the heavy price we pay – environmentally, socially and in health terms – for our high-pressure, competitive economies.

Events such as the murder of George Floyd in the US, the Black Lives Matter movement and the horrific inequitable outcomes of the pandemic on many racialized and socio-economically deprived communities afford time to pause and reflect on the shortcomings of our policy approaches. Whether or not apocryphal, hopeful or cynical, the empirical evidence in the field of policy research is clear: emergencies and disasters inspire change. This notion of significant, limited moments of disruption and potential change emerges from John Kingdon’s Multiple Streams work (4), and so-called ‘punctuated equilibrium’ thinking (5).

We call for a health promotion that pushes the envelope on promoting policies for health, well-being and equity: we want to reflect on how joined-up government and Health in All Policies can be rethought to adequately address the inequities that health promotion seeks to overcome. With the syndemic, and the 70-year history of the field we call health promotion, comes the question: ‘what are the contours and pressing issues of the health promotion we aspire to for future generations?’

IUHPE2022 convenors have identified three themes to help us reflect on the current situation of health promotion; what we do well and what we can aspire to better understand, engage in and change.

We have framed these themes as follows:

- Breaking news (the promise and opportunities for disruptions and tipping points, whether from pandemic health challenges, climate change, geopolitical shifts, social unrest or technological promise)
- Breaking free (from world-views that favor only market solutions, divisions between North and South, toward emancipatory decolonizing practices and knowledge systems)
- Breaking through (disciplines, silos, boundaries and identities engrained in our practices and understandings for innovation)

The conversations we aim to have at our conference should be engaging, even artful and full of humor. They should be inspiring, but also challenging. We recognize that the tens of thousands of – individual and institutional, card-carrying or not – members of the global health promotion community are a diverse lot. What may be challenging to some may be comforting to others. What is standard practice in the South may be radical innovation in the North. This piece intends to set some common ground for us all.

## Breaking news: global disruption

While the term ‘disruptive innovation’ stems from a business theory (6) firmly entrenched in the neoliberal economies that health promotion questions, the term has nonetheless taken on a life of its own, turning the focus around ‘turning point’ events that re-configure power relationships and the foci of agendas. In a recent series of blogs, the BMJ identified 19 global health disruptors (7). These included ravaging disease outbreaks (AIDS; severe acute respiratory syndrome (SARS); Ebola; non-communicable diseases (NCDs)), very large geopolitical events (the end of the cold war, the Framework Convention on Tobacco Control and the Belt and Road Initiative), shifts of significant prominence (urbanization; migration; climate change), and new actors and phenomena (the medical-industrial complex and the influence of large private/NGO donors).

Today more issues are recognized as disruptors: neoliberalism; Fridays for Future, COVID-19, Wet’suwet’en Strong, Marches for Justice; and Black Lives Matter. There is renewed global attention to (health) equity and its critical pathways, including colonialism and racism. However, health promotion continues to be largely politically and ecologically blind (claiming to be ‘value free’), focused almost entirely on individual or interpersonal rather than ecological determinants of health. Health promotion also struggles with meaningfully addressing continuing inequities that dog our societies. While the World Health Organization (WHO) report on the Social Determinants of Health (8) paved a path for focusing on inequities in power in order to overcome social inequities in health, we need new, better and impactful ways to address these issues through research, practice and policy.

We see ‘Social Determinants’ starting to take the path of ‘Alma Ata Primary Health Care’. Mills (9) actually predicted the patterns we have witnessed over the last decades: rather than politically engaging community assets for better primary health (which was the very intent of the Declaration), a technocratic and medical-clinical casting of the theory and practice of primary health seems to have taken it away from the people. The Social Determinants approaches are becoming technocratically dominated exercises with emphases on metrics and economic accountabilities, whereas the core intent of the program was – and remains – a socio-political one. The same seems to happen to the emancipatory potential of the Sustainable Development Goals (SDGs). These disruptions also offer opportunities of entrenchment of current systems, as previous food disruptions prior to COVID-19 have resulted in the proliferation of industrial production and trade, instead of food sovereignty (10). This makes it even more important than anticipated to organize against such entrenchment of systems that negatively shape human health. The framing of the above disruptors has turned the magnifying glass onto the relationships between these various events: connections made between the climate crisis, rights of Indigenous peoples, wealth concentration and racialized violence. The events themselves are disruptors, but we may fail to recognize that the connections between the events may be even more disrupting and require policies that cut across (or connect) disruptors.

This may be the perfect wave for (surf loving?) health promoters. It allows us to help connect the dots between these issues and turn the spotlight onto health and well-being in all policies. The disruptors identified in the BMJ piece have and will shape what global health governance does (from epidemics to climate-change refugees), how (from vaccination campaigns to commercial trade agreements) and with whom (from traditional state actors to private foundations and social movements). Yet global change and governance have local and community dimensions – and the engagement between levels and jurisdictions is critical for the identification of systems (i.e., policy and institutional) change. For instance, cities (should) aim to redesign their built environment to improve the air quality, walkability, housing, thermal comfort and sociability for all, and especially for those who live with the consequences of accumulating inequity. States (should) seek ways to improve access to health and social care for the most deprived. Health and well-being are the glue that help connect the dots between disruptors as they all translate into worsened health outcomes and increased health inequities. For health promoters this means building more health, well-being and equity into other policies, by engaging with actors who have different problems at heart, like environmental stakeholders, urban planners, social activists, infrastructure industries, etc. The conference will provide ample opportunity to learn how health promoters have worked with dedicated professionals from different policy areas. In fact – the conference may show that health promotion can very well live outside the realm of the health sector altogether.

## Breaking free: decolonizing our health practices, systems, research and policy

The second sub-theme offers alternative ways of thinking about and working in health promotion, and follows from the 2019 *Waiora – Indigenous Peoples’ Statement for Planetary Health and Sustainable Development*. This statement, developed at our last world conference, called on the global health promotion communities to make space for, and privilege Indigenous peoples’ voices and knowledge in taking action to heal our relationship with all beings of Mother Earth and focus on sustainable development. Centuries of empire-building expansion have created systems and institutions that shape widespread, systemic and on-going economic, social and health injustice. In particular, Indigenous peoples around the world continue to suffer disproportionately – culture, family ties, sustainability, ecology and knowledge systems have been deliberately and clandestinely destroyed. Health inequities are therefore products of long-term, systematic oppression of Indigenous peoples and their ways of knowing (including their ways of promoting health).

Yet decolonizing health promotion extends beyond a unique focus on Indigenous peoples. It requires creating spaces for different epistemological traditions that frame the way we see the world, the way we organize ourselves in it, the questions we ask and the solutions we seek. As we integrate other epistemologies, we recognize the importance of meaningfully working together with those who have often been ‘the studied’ to engage in the research for everyone’s benefit using participatory and community-controlled research approaches. Such participatory approaches require us to reflect on our positionality in research and reflect on ways that we can elevate community voices, needs and priorities as allies (11). *Waiora* also helps us understand some of the problems with the current neoliberal ideology and, more broadly, our capitalist ideology and system that focuses on resource extraction and individual accumulation of wealth, rather than responsibilities and reciprocity.

Erondu *et al*. (12) recently affirmed, when examining a prominent public health institution, that ‘Colonial legacies and neo-colonialism — defined by some academics as the practice of reinforcing colonialist practices of control and influence through mostly unconscious actions, behaviors, attitudes and beliefs — are the foundations of a systemic operating model that shapes career opportunities, research partnerships and teaching practices’. This colonial – or ‘foreign’ (13) – gaze is pervasive and not just an enduring legacy of the imperial ambition of a few Northern white powers. It is more insidious than that, and extends to the dominance of a particular – Cartesian – knowledge system. Mweemba *et al*. (14) demonstrate how the systemic and systematic underrepresentation of the Global South maintains an illusion of colonial superiority – even though ‘colonies’ as entities are something mostly of the past. ‘Decolonisation’, therefore, is not merely the recognition of, and apology for, a white capitalist paradigm. It is also about the de-centring of whiteness, using racial equity tools and taking the de-coloniality discourse to the South.

To decolonize health promotion and develop more effective and culturally safe health policies and programs, communities must be meaningfully driving the policy process. True engagement and participation need to be secured. We must challenge the notion that research findings from Western mainstream societies (the Global North) are directly applicable in other contexts. Instead, we must generate knowledge in, with and for Indigenous and minority communities to promote health equity. Research involving Indigenous researchers and community members is needed to bridge and close the divide (15). Such decolonizing research processes show the path to co-create intelligence and shift power dynamics to support profound innovation and radical change. The health promotion toolkit should embrace innovations in Indigenous research methods such as storytelling, Dadirri and Two-Eyed Seeing (16).

## Breaking through: emancipatory innovation

In the lead-up to IUHPE2022, the global health promotion community (including IUHPE and other institutions, but also policy makers, activists and critical institutions) needs to identify key innovations with the potential to change ways of thinking about problems and their solutions. We need to start identifying the individuals, communities and their networks that can drive change – at policy and systems levels. Innovation often starts small and takes time to diffuse. Its success, however, comes about through networking for its discovery, acknowledgment and dissemination. IUHPE2022 must allow this.

Old innovations (such as Artificial Intelligence) must be refreshed with a potent health promotion, well-being and equity lens (such as embracing the Montreal Declaration for a responsible development of Artificial Intelligence (17) at IUHPE2022). Similarly, the mobilization of social movements for equity and well-being is already part of our repertoire. Whether we always do this well, or accountably, is worth critical examination. Global (social media) networking and engagement create new opportunities for more, if not all, voices to be heard. Inspired leadership and ‘Learning by Doing’ (18) must become integral to policy change.

Another field of innovation in health promotion is a more significant and deliberate framing of power systems and interests that drive the maintenance of ways of working, doing and arranging the ‘who gets what, why and when’ matters of the political game. This is the very essence of health promotion, and apart from some persistent ideologues on the fringe, our movement has been unable to integrate novel ideas such as econology (19), the consucracy (20), transformative intergenerational change and polarizing value systems in a strong action agenda.

We break (news; free; and through) in different ways and invite you to come together on the Haudenosaunee/Anishinaabe traditional territory of Tiohtià:ke (Montreal) in May, 2022. The disruption of the syndemic has created opportunities for a hybrid (in-person and virtual) conference that allows for many more voices to be heard and more minds to be brought together. Help us to continue fruitful disruptions, decolonize our joint global commons, and innovate for better health, well-being and equity. We invite health promoters, communities, activists, scholars and, most importantly, policy operators to help transform our world for the benefit of all Nations and all our relations with Mother Earth.
